# The AnimalAssociatedMetagenomeDB reveals a bias towards livestock and developed countries and blind spots in functional-potential studies of animal-associated microbiomes

**DOI:** 10.1186/s42523-023-00267-3

**Published:** 2023-10-05

**Authors:** Anderson Paulo Avila Santos, Muhammad Kabiru Nata’ala, Jonas Coelho Kasmanas, Alexander Bartholomäus, Tina Keller-Costa, Stephanie D. Jurburg, Tamara Tal, Amélia Camarinha-Silva, João Pedro Saraiva, André Carlos Ponce de Leon Ferreira de Carvalho, Peter F. Stadler, Danilo Sipoli Sanches, Ulisses Rocha

**Affiliations:** 1 Department of Environmental Microbiology, Helmholtz Centre for Environmental Research - UFZ GmbH, 04318 Leipzig, Germany; 2 Department of Computer Science and Interdisciplinary Centre of Bioinformatics, University of Leipzig, Härtelstraße 16-18, 04107 Leipzig, Saxony, Germany; 3 Institute of Mathematics and Computer Sciences, University of Sao Paulo, Sao Carlos, Brazil; 4 GFZ German Research Centre for Geosciences, Section 3.7 Geomicrobiology, 14473 Telegrafenberg, Potsdam, Germany; 5 Federal Univ. of Technology - Paraná (UTFPR), Cornélio Procópio, Brazil; 6 Institute for Bioengineering and Biosciences (iBB) and Institute for Health and Bioeconomy (i4HB), Instituto Superior Tecnico (IST), Universidade de Lisboa, Lisbon, 1049-001 Portugal; 7 Department of Bioanalytical Ecotoxicology, Helmholtz Centre for Environmental Research - UFZ, Leipzig, Germany; 8 Hohenheim Center for Livestock Microbiome Research (HoLMiR), University of Hohenheim, Stuttgart, Germany; 9 Institute of Animal Science, University of Hohenheim, Stuttgart, Germany; 10 German Centre of Integrative Biodiversity Research (iDiv) Halle-Jena-Leipzig, Puschstraße 4, Leipzig, 04103 Germany; 11 Max Planck Institute for Mathematics in the Sciences, Inselstraße, 04103 Leipzig, Germany; 12 Institute for Theoretical Chemistry, Universität Wien, Währingerstraße 17, Vienna, A-1090 Austria; 13 Center for Scalable Data Analytics and Artificial Intelligence Dresden-Leipzig, Leipzig University, Leipzig, Germany; 14 Faculdad de Ciencias, Universidad Nacional de Colombia, Sede Bogotá, Bogotá, Colombia; 15 Center for non-coding RNA in Technology and Health, University of Copenhagen, Frederiksberg, Denmark; 16 The Santa Fe Institute, 1399 Hyde Park Rd., Santa Fe, NM 87501 USA

**Keywords:** Metagenome, Animal-Associated Microbiomes, Microbial Ecology, Metadata, Database, FAIR principles

## Abstract

**Background:**

Metagenomic data can shed light on animal-microbiome relationships and the functional potential of these communities. Over the past years, the generation of metagenomics data has increased exponentially, and so has the availability and reusability of data present in public repositories. However, identifying which datasets and associated metadata are available is not straightforward. We created the Animal-Associated Metagenome Metadata Database (AnimalAssociatedMetagenomeDB - AAMDB) to facilitate the identification and reuse of publicly available non-human, animal-associated metagenomic data, and metadata. Further, we used the AAMDB to (i) annotate common and scientific names of the species; (ii) determine the fraction of vertebrates and invertebrates; (iii) study their biogeography; and (iv) specify whether the animals were wild, pets, livestock or used for medical research.

**Results:**

We manually selected metagenomes associated with non-human animals from SRA and MG-RAST.  Next, we standardized and curated 51 metadata attributes (e.g., host, compartment, geographic coordinates, and country). The AAMDB version 1.0 contains 10,885 metagenomes associated with 165 different species from 65 different countries. From the collected metagenomes, 51.1% were recovered from animals associated with medical research or grown for human consumption (i.e., mice, rats, cattle, pigs, and poultry). Further, we observed an over-representation of animals collected in temperate regions (89.2%) and a lower representation of samples from the polar zones, with only 11 samples in total. The most common genus among invertebrate animals was Trichocerca (rotifers).

**Conclusion:**

Our work may guide host species selection in novel animal-associated metagenome research, especially in biodiversity and conservation studies. The data available in our database will allow scientists to perform meta-analyses and test new hypotheses (e.g., host-specificity, strain heterogeneity, and biogeography of animal-associated metagenomes), leveraging existing data. The AAMDB WebApp is a user-friendly interface that is publicly available at https://webapp.ufz.de/aamdb/.

**Supplementary Information:**

The online version contains supplementary material available at 10.1186/s42523-023-00267-3.

## Background

Metagenomics is an expanding field of study, and the number of metagenomes in public databases has grown exponentially [1]. While genomic studies consider a specific organism’s genetic material, metagenomic studies consider the genetic material of entire communities of organisms [2, 3]. They have served to inform a wide range of fields, including Earth Sciences, Life Sciences, Biomedical Sciences, Bioenergy, Bioremediation, Biotechnology, Agriculture and Biodefense, and Microbial Forensics [4]. Metagenomic data is generally deposited in public sequence repositories, the largest of which is the Sequence Read Archive (SRA) [5] of the National Center for Biotechnology Institute (NCBI) [6], which is associated with the International Nucleotide Sequence Database Collaboration (INSDC) [7], a collaboration between the DNA Databank of Japan (DDBJ) [8], and the European Nucleotide Archive (ENA) [9]. Smaller repositories for metagenomic data include MG-RAST [10], IMG/M [11], MGnify [12], and gcMeta [13]. However, identifying relevant studies is not straightforward. As the number of publicly available metagenomic studies continues to grow, developing centralized resources and curated metadata is essential to improving the findability and reusability of these data. While several such resources exist for environmental [1, 14, 15] and human-associated metadata [16–19], animal-associated metagenomes have received less attention. Searching and reusing samples can be difficult due to incomplete or poor-quality metadata accompanying the metagenome data [14–16, 20–22].

Databases allow for the re-analysis of samples to test new hypotheses and make novel discoveries [23]. For instance, a study by Stewart and collaborators [24] assembled 4,941 rumen microbial metagenome-assembled genomes (MAGs). Reusing metagenomes in public repositories may lead to new genomic and protein resources, enabling a better understanding of the structure and functions of the (in this case rumen) microbiota. This highlights the importance of creating user-friendly metagenome repositories as the starting point for meta-studies of animal microbiomes. Nevertheless, mining metagenomes of interest from public databases is time-consuming and requires specialist knowledge in bioinformatics and microbiology, as metadata are not easily accessible for those with little experience in data science [25]. Some initiatives try to simplify this process, such as the Genomics Standard Consortium [26], the BioProject, and the BioSample project [27], which defined the minimum necessary information about a metagenomic sample to facilitate and provide better organization of metadata [28].

Nevertheless, tools are still needed to filter samples based on metadata in a user-friendly way. For example, the HumanMetagenomeDB contains standardized metadata of about 70,000 human metagenomes [16]. Further, the TerrestrialMetagenomeDB contains curated metadata of more than 20,000 terrestrial metagenomes [14], and the MarineMetagenomeDB over 11,449 marine metagenomes [15]. Recently, Hu and collaborators [20] developed an animal metagenome database that contains 10,672 publicly available metagenomes and 63,214 amplicon sequencing samples from animal-associated microbiomes and divides the data into domestic and wild animal categories. Users can download the metadata of interest according to filters but cannot download raw data or visualize the distribution of selected data on a world map. Furthermore, an analysis of data distribution across different species and regions is not available. Research into animal-associated metagenomes could be significantly accelerated by developing attributes tailored for non-human, animal-associated data and presenting a user-friendly catalogue of available data.

Improved data accessibility can shed light on current data collection biases. One study demonstrated a higher data collection from temperate zones and vertebrate animals in biodiversity studies [29]. Indeed, most research on biodiversity and microbiomes happens in countries with larger economies [30]. At the same time, up to 50% of all species on Earth may be native to 6–7% of the Earth’s land area that is covered by tropical moist forests [31]. Many developing nations are located in the tropics, where the level of biological diversity is the highest, and the threats to its maintenance are the greatest [32]. Further, while it is estimated that 925,000 species of invertebrates [29] and 100,000 species of vertebrate animals [33] inhabit Earth, biodiversity research shows a general bias towards vertebrates [29]. Identifying gaps is essential, especially in the young and rapidly growing field of metagenomic research. Awareness alone may aid in curving these biases in research as they develop [29]. In addition, biodiversity loss and species extinction are two critical environmental problems. Microbiome-targeted interventions have been studied as potential options to reverse the deterioration of biodiversity [34].

To tackle the challenges of the non-standardization and ambiguity of the metadata, we developed the AAMDB, which has standardized and manually curated animal-associated metadata to help researchers quickly identify animal-associated metagenomes of interest through a user-friendly web interface. Further, we used this resource to evaluate bias in the distribution of animal-associated metagenomes, indicating which are the most studied and the under-represented species in public repositories.

## Implementation

**Fig. 1:**
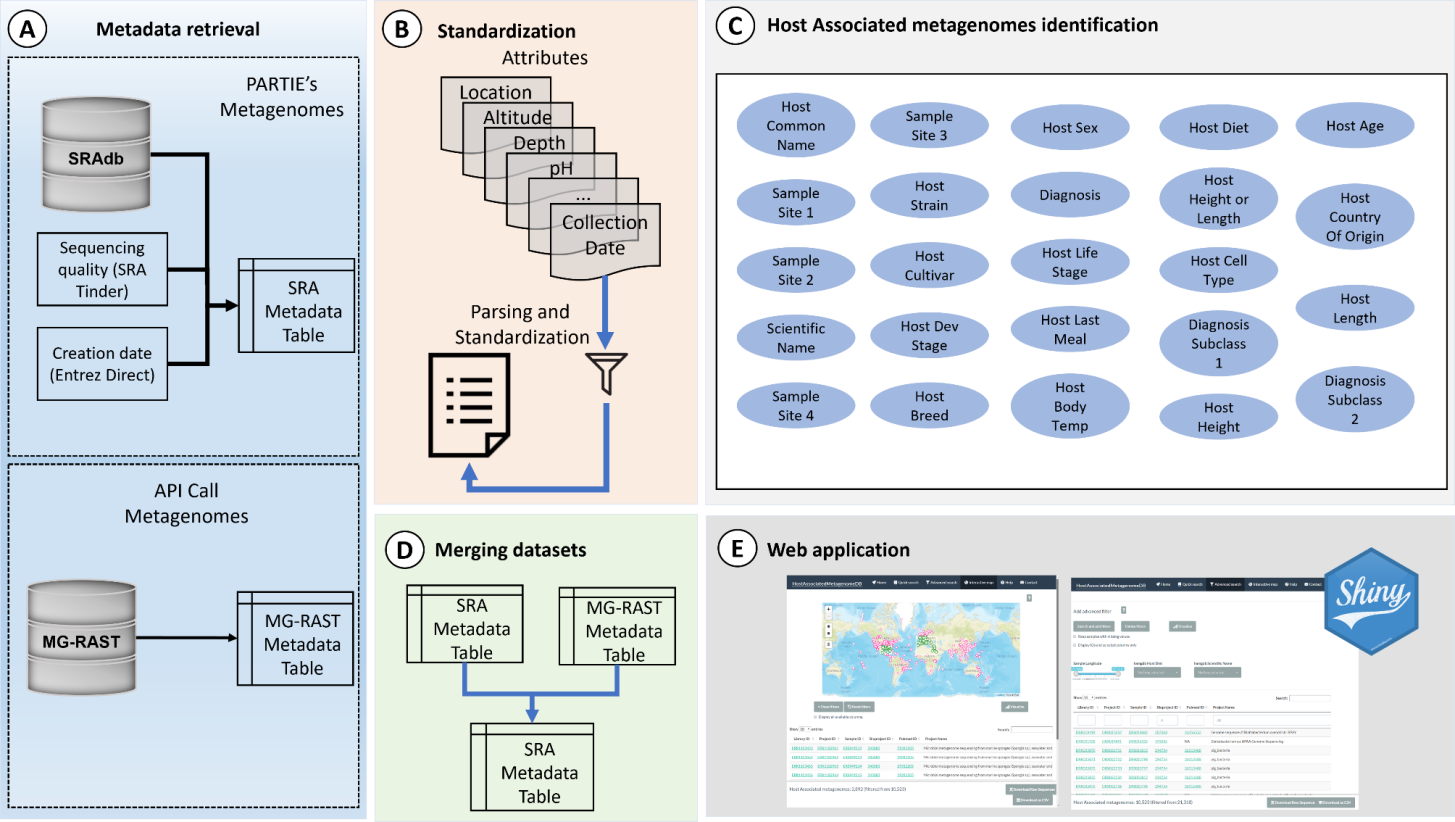
Overview of the AAMDB construction workflow. (**A)** Metadata retrieval present in SRA and MG_RAST; (**B**) standardization of attributes; (**C**) identification of animal-associated metagenomes terms; (**D**) merging SRA and MG_RAST dataset; (**E**) The AAMDB was made available through a Shiny web application

**Fig. 2:**
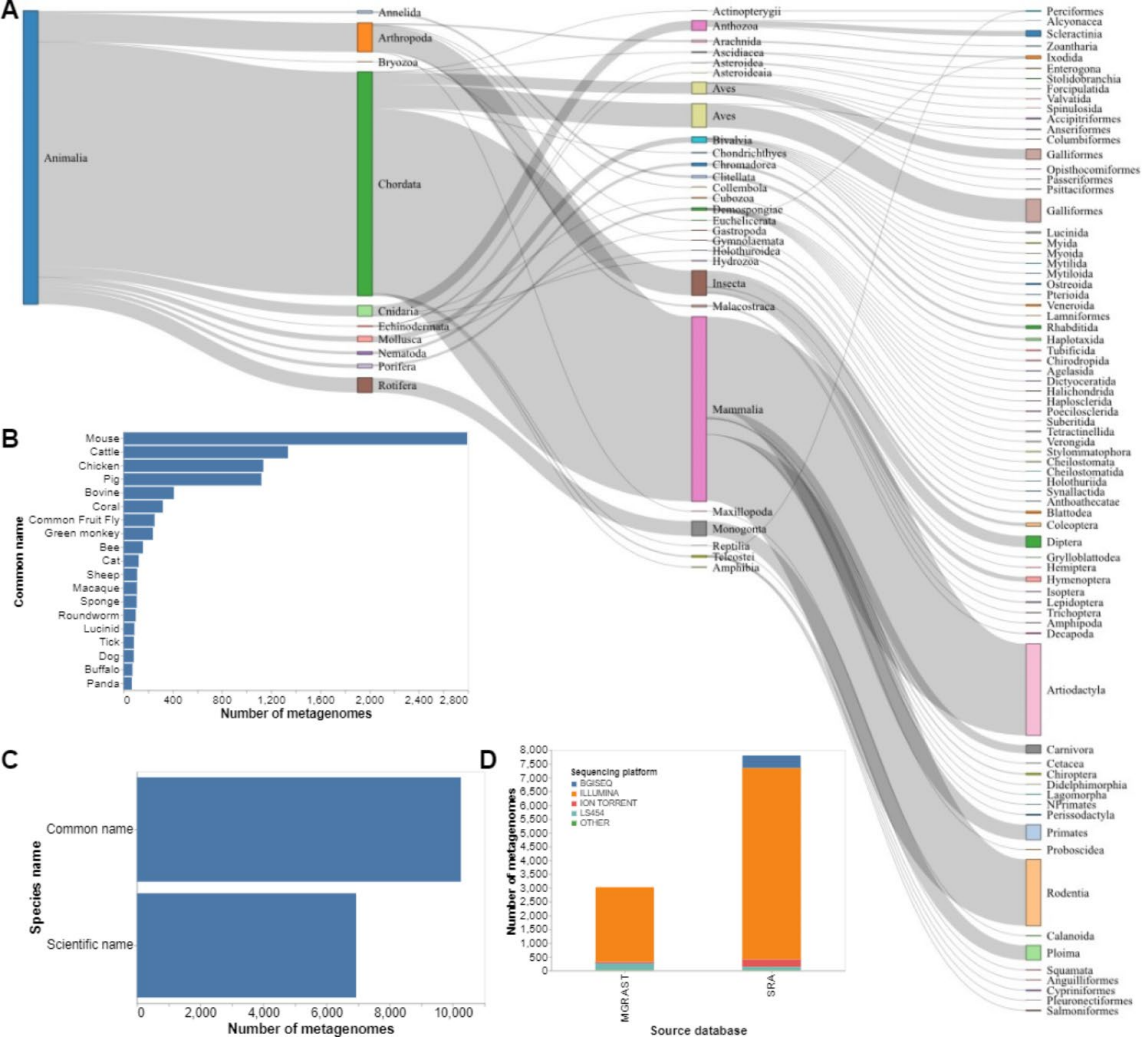
Descriptive statistics of the AAMDB content. (**A**) Sankey plot containing the taxonomic classification in Kingdom, Phylum, Class, and Order. (**B**) Bar plot of the distribution of the top twenty (20) common host animal names. (**C**) Bar plot of the distribution of the samples with the host animal species name. (**D**) Bar plot of the distribution of sequencing technologies (Sequencing platform) per database of origin (Source database)

**Fig. 3:**
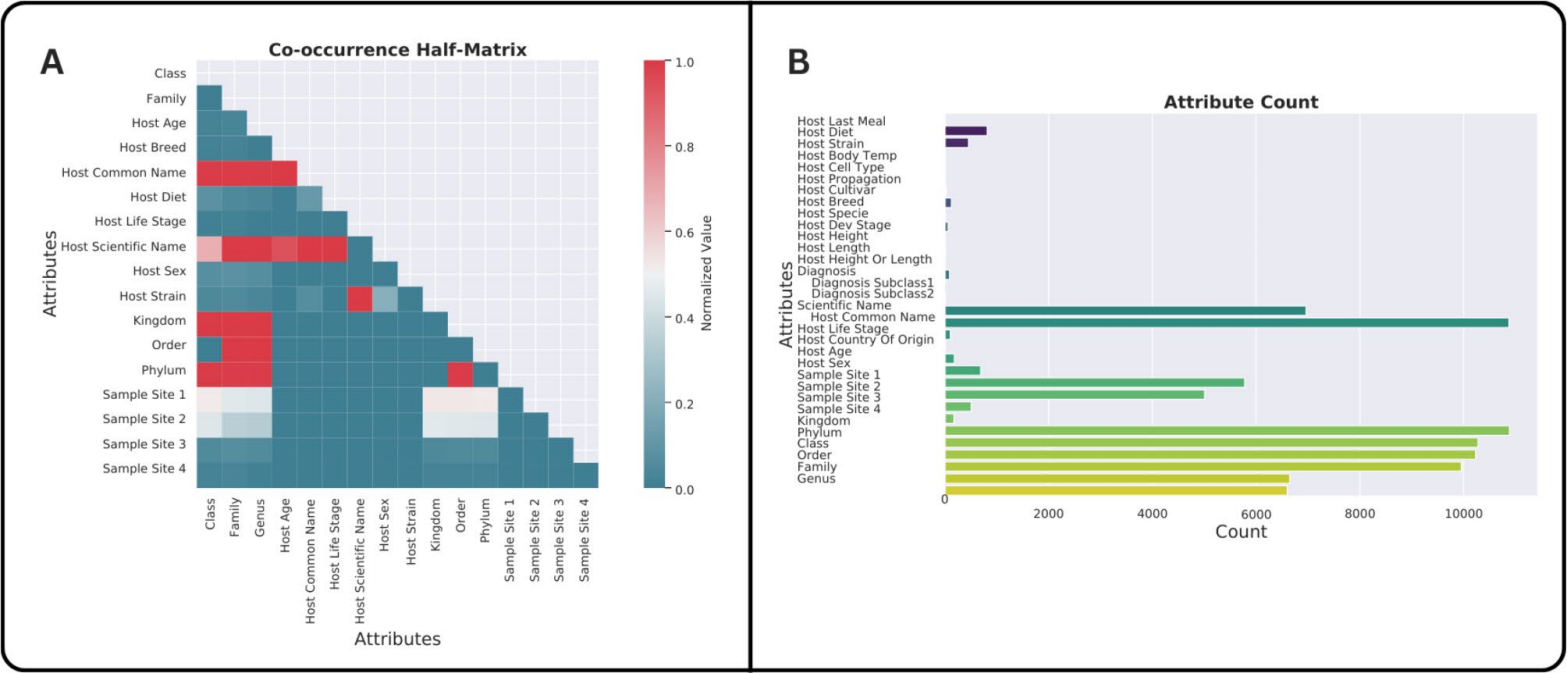
Co-occurrence relationships and prevalence of attributes. (**A**) Half-matrix visualization providing a detailed representation of the frequencies of animal-associated attributes, capturing the complex co-occurrence relationships among different attributes. (**B**) Bar chart display showcasing the occurrence count of the predominant attributes, broken down per individual sample. This analysis illustrates the distribution and prevalence of these attributes within the collected samples

**Fig. 4:**
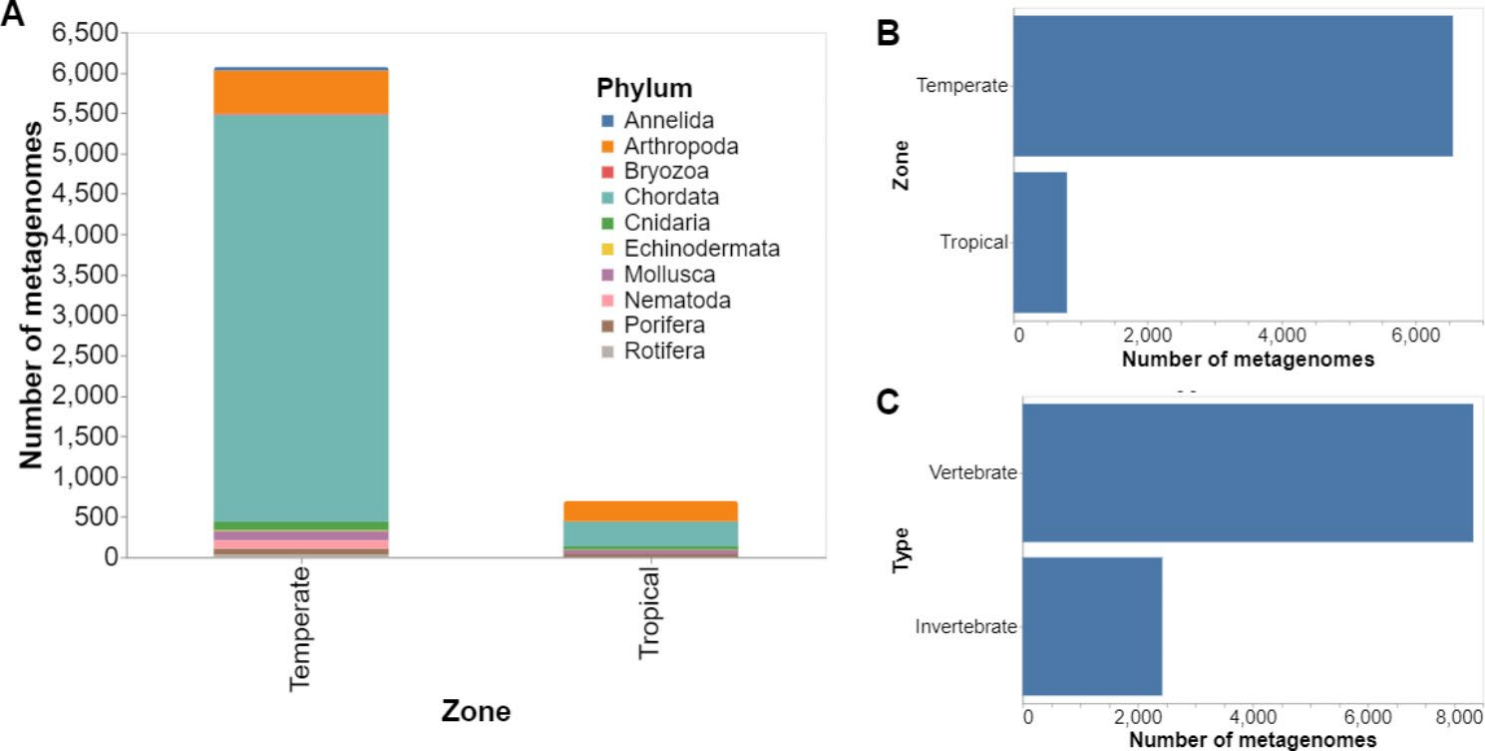
Bias in sampling animal-associated metagenomes. (**A**) Bar Plot of the number of metagenomes by host phylum divided by tropical and temperate zone. (**B**) Bar plot of the samples’ distribution in the tropical and temperate zone. (**C**) Bar Plot of the distribution of microbial metagenome samples from vertebrate and invertebrate hosts

We constructed the AAMDB in three steps (Fig. 1): (1) metadata retrieval from the source databases (SRA and MG-RAST) and removal of human and non-animal metagenomes (Fig. 1A); (2) selection, parsing, and standardization of available metadata attributes (Fig. 1B); (3) identification of animal-associated metagenomes terms (Fig. 1C); (4) merging datasets (Fig. 1D); and (5) development and implementation of a user-friendly web application (Fig. 1E).

At different points of the implementation, we will use the expression ‘manually curated’. Any manual process has risks and setbacks, such as human errors, biases, and limitations, so it cannot be considered inherently superior to an automatic process. In this study, manual curation was done by specialists in the field who identified issues in terminology, standardization and usage of different types of metadata. Once issues were identified, we generated automated or semi-automated scripts to address them, minimizing the risks and setbacks.

### Metadata retrieval and removal of non-whole genome sequencing data

Metadata from SRA were retrieved from SRAdb using a list of sample identifiers (SRA run IDs) labeled as whole-genome sequencing from complex microbial communities (hereafter, metagenomic data) or amplicon sequencing was downloaded from PARTIE (Ref = Torres, Edwards, e McNair, “PARTIE”) (https://github.com/linsalrob/partie). PARTIE is a Machine Learning model based on supervised and unsupervised classification, which classifies sequence data into metagenomic or amplicon sequence data sets. Sample identifiers labeled as WGS were extracted from the list, and metadata of WGS samples was retrieved using SRAdb R package. This package provides access to metadata of samples available in SRA. We retrieved quality scores and the creation date of the SRA libraries using SRA-Tinder (https://github.com/NCBI-Hackathons/SRA_Tinder) and Entrez Direct (https://www.ncbi.nlm.nih.gov/books/NBK179288) respectively. Finally, we recovered the PubMed and BioProject ID using the rentrez tool (https://github.com/ropensci/rentrez). Concurrently, all metadata from the MG-RAST repository were retrieved using their application program interface (API).

Once the metadata were downloaded, we removed non-whole genome sequencing (non-WGS) from SRA and MG-RAST. For SRA, the non-WGS samples were eliminated by removing samples that contained ‘AMPLICON’ or ‘*RNA*’ in the ‘library_strategy’ category. We also removed samples labeled ‘*PCR*’ in the ‘library_selection’ category. After, we removed all samples labeled with anything other than ‘METAGENOMIC’ or ‘GENOMIC’ as their ‘library_source’ and manually checked for entries belonging to single species genomes. We removed all samples marked with anything other than ‘WGS’ for MG-RAST as in the categories ‘investigation_type’ and ‘seq_meth’.

### Selection of non-human animal-associated metagenomes, parsing, and standardization of attributes

To select animal metagenomes, the column ‘sample_attribute’ was manually explored, and host names were extracted, and a dictionary of terms containing the host names and human-related terms was created (Supplementary Table S1). The columns ‘center_project_name’, ‘sample_attribute’, and ‘study_title’ were extracted, as they contained information on the source of the sequence data. Then, a vector with the flags ‘keep’, ‘remove’, or ‘NA’ was created for each metadata column (Supplementary Table S2). For each one of the three columns, samples were labeled ‘keep’ when they contained non-human, animal host names, ‘remove’ when they contained human terms, or both terms, human and non-human hosts term or ‘NA’ when they contained none of the terms in the dictionary and samples marked with only ‘remove’ or ‘remove’ and ‘NA’ were eliminated, while samples marked with either only ‘keep’ or ‘keep’ and ‘NA’ were retained. Samples were manually curated when they contained both ‘keep’ and ‘remove’, while samples marked with only ‘NA’ were labeled ‘undefined’. We extracted the column ‘study_abstract’ to determine, case by case, whether samples classified as ‘check’ and ‘undefined’ were non-human host-associated. We manually inspected all samples after that to confirm their suitability.

In SRAdb, all the sample features are found in a single field named ‘sample_attributes’. Therefore, the feature names and values of the samples in the ‘sample_attributes’ field are not coherently organized into distinct and well-defined metadata categories. We parsed the field attribute names and determined their frequency of occurrence. We removed sample attribute names with less than ten occurrences across a single dataset as they would make manual curation impossible. Next, we manually grouped synonymous attribute names (Supplementary Table S3).

Further, we extracted and standardized the values of ten attributes: sample altitude, sample elevation, sample collection date, sample temperature, sample pH, sample salinity, sample depth, sample latitude, sample longitude, and sample location (country and ocean/sea). Dates were standardized using International Standard Organization (ISO) 8601 (YYYY-MM-DD) [35]. Sample latitude and longitude were standardized to the format of decimal degrees. Location (country) was manually labeled following the standard of ISO 3166-1 [36]. The hosts’ common and scientific names were derived from the metadata. The taxonomy of the hosts was identified using the R package ritis (Integrated Taxonomic Information System Client) [37]. Samples with no scientific names in the metadata were treated manually, and the following actions were taken sequentially: (a) their scientific names were searched in different taxonomy browsers on the internet (i.e., NCBI Taxonomy [38] and ITIS (Integrated Taxonomic Information System) [39]); (b) the taxonomic level of the common name was identified; (c) the complete taxonomy string of the host was deduced; (d) lastly, all the taxonomic levels are marked as NA.

We also identified and standardized four categories of host attributes: host characteristics (e.g., age, sex, height, length), host exposure (diet and last meal), host diagnosis, and sampling site/material. Host ages were standardized to years, length and height were standardized to a uniform SI system unit, and sex was standardized based on the metadata in the respective repositories. The sampling sites of mammals were organized into eight main categories: ear, gut, liver, lung, nose, oral, skin, biofluid, and the whole organism. We used the BRENDA [40] Tissue and Enzyme Source Ontology (http://bioportal.bioontology.org/ontologies/BTO/?p=classes&conceptid=root) to homogenize the ‘Sample_Site’ attributes. Terms used during the standardization of the attributes can be found in Supplementary Table S3.

For MG-RAST, the selected attributes were mostly already standardized, e.g., ‘project_name’, ‘seq_meth’, ‘latitude’, and ‘country’, among others. Therefore, the columns were adapted to the standard created during the standardization of the SRA retrieved metadata. The kingdom of the hosts was used to eliminate non-animal hosts. The complete set of standardized attributes can be found in Supplementary Table S4.

### Combining SRA and MG-RAST

We identified equivalent and comparable attributes from the curated SRA metadata with those in metadata provided by MG-RAST (Supplementary Table S5), and the two metadata tables were merged. Five attributes (three related to library sequencing quality and two to sample attributes) were specific to SRA and MG-RAST, respectively. They are; ‘quality_above_30_SRA’, ‘mean_quality_SRA’, ‘sample_pH’, ‘sample_salinity’, for SRA and ‘drisee_score_raw_MGRAST’ for MG-RAST.

### Web app implementation

The AAMDB web application was implemented using Shiny (version 1.5.0), an R package (version 3.6.3). The app was designed with a tab layout. The tabs include. ‘Home’, ‘Quick search’, ‘Advanced search’, ‘Interactive map’, ‘Help’, and ‘Contact’. The ‘Home’ tab steers users around the application. The tabs ‘Quick search’ and ‘Advanced search’ provide filter options to aid users in selecting samples of interest. The ‘Interactive map’ tab allows users to select samples based on location. The interactive map functionality was implemented using the leaflet package (version 2.0.3), and we implemented the toolbox for selecting areas on the map with geoshaper (version 0.1.0) and the sp packages (version 1.4-2). The remaining R packages and their respective versions can be found in Supplementary Table S6 (Supplementary Material 6) [41–152]. The web application is available at https://webapp.ufz.de/aamdb/.

### Quick search

The ‘Quick Search’ tab gives users access to all AAMDB content, and it is also possible to filter by main attributes. This feature shows all metagenomes, including those that do not have a valid geographic coordinate. The WebApp allows users to set up input filters using 20 available filters or by typing in the search box at the top of the table. After filtering the metadata, one can download a sample table with the associated metadata as comma-separated values (.csv) file. The metadata of the entire dataset can be downloaded if the user does not apply any filter. The steps to obtain raw sequencing data are described below.

### Advanced search

The ‘Advanced Search’ tab allows the creation of a dynamic filter for the available attributes. We implemented a checkbox to allow users to filter out samples with missing values for the chosen attributes. The user can click the ‘Search and add filters’ button and a window will open. Searches for attributes can be made by name. Further, they are also organized using the following categories: ‘Sample Attributes’, ‘Host Characteristics’, ‘Host Identity’, ‘Sample Site’, ‘Sample Location’, ‘Sequencing Features’, ‘Host Diagnosis’, and ‘Host Exposure’. At the top of the ‘Advanced Search’ page, we added a dropdown menu where users may select attributes to be added in the table below. After applying the filters and associated values, the metadata of selected entries can be downloaded as a comma-separated values (.csv) file.

### Interactive map

The ‘Interactive map’ tab allows users to find the samples according to their location on the world map. Only samples with valid coordinates can be selected on the map. We implemented drawing tools (e.g., rectangular or polygon shapes) to help users select samples on the map.

It is important to note that individual points marked on the map may represent more than one sample since multiple samples can come from the same coordinate position. After selecting samples on the map, the selected metagenomes will be shown in the data table below the map. The users can further filter the samples using the ‘Show filters’ option, which will open the ‘Quick Search’ tab to filter the samples. After filtering the dataset, the resulting metadata table can be downloaded as a comma-separated values (.csv) file.

### Downloading the raw data from selected metagenomes

We developed a simple download procedure to obtain raw sequence data from SRA. Unfortunately, MG-RAST does not allow public downloads anymore. Our Python scripts enable simple installation of the SRAtoolkit (https://trace.ncbi.nlm.nih.gov/Traces/sra/sra.cgi?view=software) and the download of specific metagenomes or all metagenomes using the table exported by the WebApp, with two user-friendly commands. To support less experienced users, a script with a graphical user interface (GUI) is available (link at the end of the paragraph). Although most users may operate on Linux systems, we provide Windows executables to allow instant execution without installation. The download scripts are additionally [AB1] compatible with the CSV exports from the TerrestrialMetagenomeDB, HumanMetagenomeDB, and the MarineMetagenomeDB and are provided at https://github.com/mdsufz/downloadtool.

## Results and discussion

### Sample distribution and under-represented species and areas

The current version of the AAMDB version 1.0 includes metadata for 10,885 animal-associated metagenomes. Among them, 7,817 (71.81%) samples were retrieved from the SRA, and 3,040 (28.19%) samples were retrieved from MG-RAST. Hosts represented in our database span 10 phyla, 28 classes, 74 orders, 122 families, 174 genera, and 283 species (Fig. 2A). Samples with information on common host names were concentrated in four host species (mouse, cattle, pig, and chicken), which accounted for 5,560 (51.1%) samples (Fig. 2B). All samples had the attribute ‘species name’ filled. We split the host species’ names into their common and scientific names. Most samples, 10,847 (99.65%), had the common host name, and 6,935 (63.71%) had the scientific host name (Fig. 2C). Illumina-based technologies were the most frequent sequencing technology, with 9,663 (88.77%) samples. BGISEQ followed Illumina data with 462 (4.24%) samples, followed by Roche LS454 (3.19%), ION TORRENT (2.93%), PacBio (0,3%) and Nanopore (0,1%) (Fig. 2D). All animal attributes’ frequency and co-occurrence were examined (Fig. 3). Beyond the differences in technology, we also analyzed the biogeography of the entries in our database regarding the following host distributions: (a) taxonomy; (b) use (domestic, wild animals, food stock and medical research); (c) aquatic vs. terrestrial animals; (d) vertebrate vs. invertebrate; (e) climate and economy of the country generating the data.

The metadata metagenome samples are distributed according to the taxonomy. There were 10,258 (94.24%) samples with a phylum assigned, 10,204 (93.74%) had a class and 9,942 (91.33%) had an order assignment. Family and genus were assigned to 6,635 (60.95%) and 6,585 (60.50%) samples, respectively. Using the taxonomic classification information from our data, we observed a bias towards vertebrate animals. Vertebrates represented 7,831 (76.34%) samples, whereas 2,427 (23.66%) were collected from invertebrates (Fig. 4C). The most common genus among invertebrates was *Trichocerca* (rotifers), with 512 samples (21% of the total invertebrate samples). Most vertebrates were used for livestock, medical research, and pets, representing 6,999 samples (64.30%, Fig. 4A). Of these samples, 2,792 (25.64%) represented mice, usually used in medical research. Livestock animals (bovine, chicken, pig) represented 4,001 (36.75%) samples while 206 (1.90%) samples represented pets. On the other hand, there were samples from wild animals such as gorillas with 43 samples, green monkeys with 238 samples, and dwarf tiger lucines with 7 samples.

Aquatic animals represented 1,017 (9.34%) entries, of which 326 (3%) were coral species and 512 (4.70%) were rotifers. The phylum Arthropoda comprised 1,010 (9.27%) samples. Other relatively frequent invertebrate groups of the dataset were mollusks. A study concerning biomass distribution on Earth [153] highlighted the impact of human civilization on global biomass through livestock. The AAMDB could be explored to examine the anthropogenic activity in animal microbiomes (e.g., following biodiversity and functional-potential profiles of wild animals, pets, and food stock over time).

**Fig. 5:**
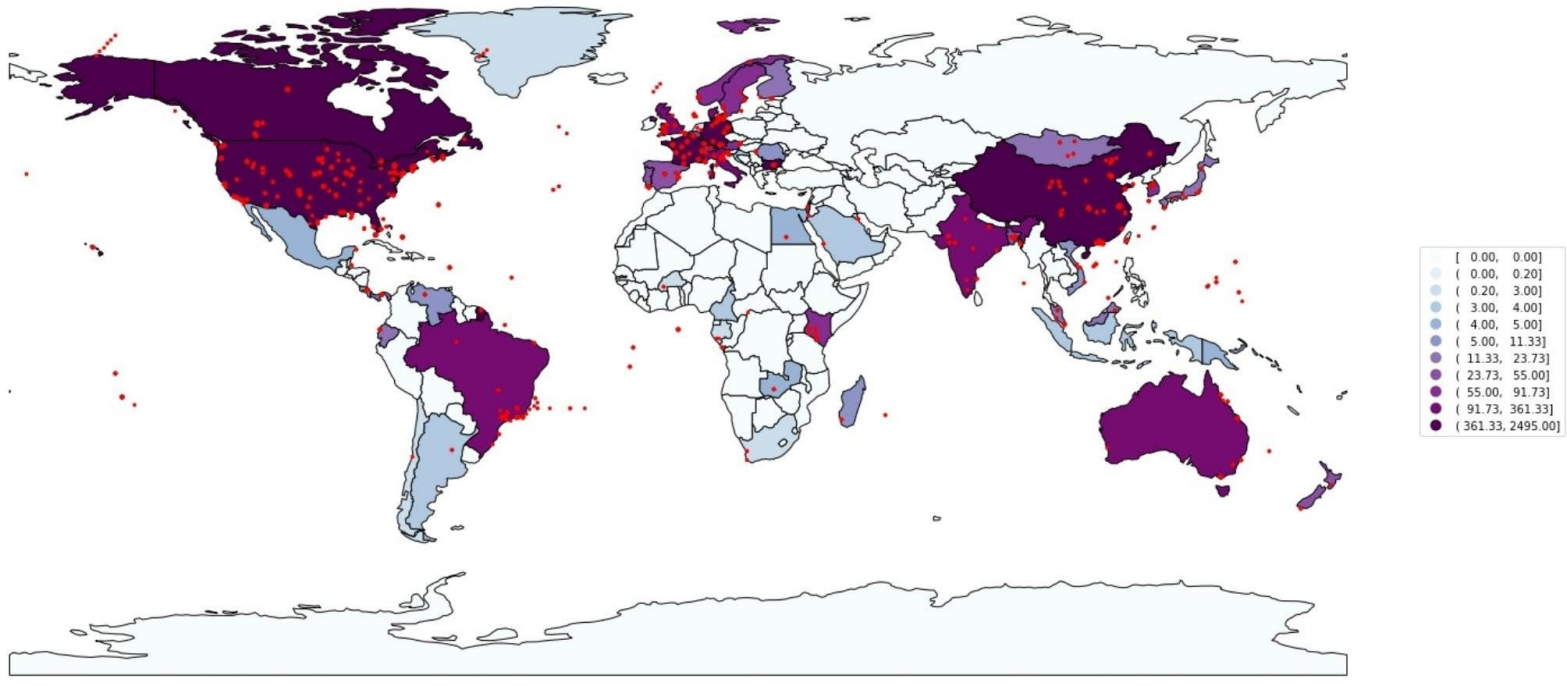
World map showing the number of metagenome samples by region/country

A total of 6,808 entries had geographical coordinates. We found that temperate biomes were more extensively sampled than tundra, flooded grasslands, savannas, mangroves, and most tropical biomes, likely reflecting differences in funding availability, access to modern molecular biology laboratories, and/or expertise in metagenomics analyses across countries [154]. In this work, 6,553 (89.16%) samples were retrieved from temperate climate zones, and only 796 (10.84%) were collected in tropical regions (Fig. 4B). Only 11 of the samples that had coordinates were from polar regions. Figure 5 shows that sampling has been concentrated in areas with higher economic indexes. However, these regions do not have the highest biodiversity [30].

To start addressing these gaps, awareness and support from the international scientific community are urgently needed for nations located in the tropics, where biological diversity is highest and the threats to its maintenance are the greatest [34]. Indeed, animal microbiomes in tropical countries are less studied than in temperate countries [10].

### Usage and functionalities

**Fig. 6:**
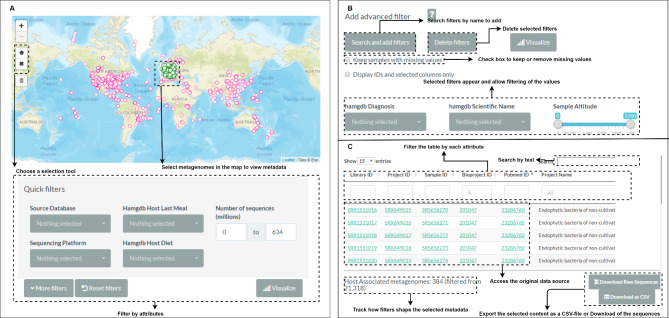
AAMDB user-interface overview. (**A**) The ‘Interactive Map’ allows users to select samples according to their geographical location on the map using a selection tool. (**B**) The ‘Advanced search’ tab allows users to select as many filters as they want, and the metadata is displayed under the filtering options

The AAMDB has a simple and user-friendly interface consisting of three main sections, which allow users to select the type of query that suits their needs (Fig. 6). The ‘Quick Search’ section allows users to apply quick filters with some attributes that appear initially. Clicking the “more filters” button allows the selection of further filters according to the user’s needs. The ‘Advanced Search’ section has filters for all dataset attributes, allowing for a dynamic combination of these attributes. Finally, the ‘Interactive map’ section provides the user with a visual and intuitive search to select metagenomes according to the geographic coordinates of the samples. This functionality is limited to metagenomes with a valid geographic coordinate. Each sample has identification attribute hyperlinks (‘sample_id’, ‘project_id’, ‘library_id’, ‘PubMed ID’, and ‘BioProject ID’) to the source database (MG-RAST and SRA). All sections include functionalities to visualize the data distribution as a pie chart with the percentages of selected data or a histogram with the data distribution selected by the user, where the X-axis represents the value for a specific attribute chosen by the user, and the Y-axis represents the number of samples with this value. These features are intended to help users better understand the distribution of the selected data after filtering and the number of samples with a given attribute value.

While developing the AAMDB, we ensured that users could filter metadata using all attributes and combine filters to optimize data searches. Indeed,  our database contains 51 attributes (e.g., Host Body Temperature, Host Characteristics, Host Diagnosis, Host Strain and Host Sex). We also provide a tool for automatically downloading metagenome samples from the SRA database (see item ‘Downloading the raw data from selected metagenomes’ later in the manuscript).

### Usage example

Bees are considered essential for maintaining biodiversity on Earth [155]. Scientists interested in bees may use the AAMDB to find metagenomes recovered from bees. The user can select the ‘More filters’ tab on the Quick Search tab to search for *bee* under ‘AAMDB Host Common Name’, resulting in a list of 157 samples. Users can select samples from countries of interest under the ‘Sample Location Country’ filter. Further, the user may select samples from ‘Switzerland’, decreasing the number of samples to 24. The user can click ‘Visualize’ to explore the selection. After the selection, the user can download the selected metadata as a CSV file for further analysis using an icon in the bottom right of the webpage and use our download tool (see the previous item) to retrieve the raw sequence data of the selected samples.

### Database update plan

The number of metagenomic experiments submitted to public repositories, like SRA, is growing rapidly. Therefore, due to the manual curation steps that are necessary to construct our database, we will update the AAMDB with newly submitted microbial metagenome samples every February. Updates will serve as opportunities to include novel features or modify existing ones. Users can send questions and suggestions using the information included in the contact tab.

### Suggestions for good practices

One of the goals of this work was to facilitate metagenome meta-analyses. To this end the AAMDB provides curated taxonomic classifications of the host animals, and includes a help guide for the community to improve metadata annotation when submitting novel metagenome samples to public repositories. Suggested ontologies can be located under Point 7 in the ‘Help’ tab of the AAMDB website under the title ‘What should I do to include my metagenomes in AAMDB?‘. Our database is not a repository for raw data. Still, when users follow our suggestions when submitting new entries to SRA, these new samples will be added to our metadata database during the yearly update.

The study by Wilkinson and collaborators [156] recognizes Findability, Accessibility, Interoperability, and Reusability (FAIR principles) as key pillars of robust research and effective data management. The AAMDB strictly adheres to these four principles. Firstly, the Findability of our data is ensured as each sample is associated with a unique identifier. The metadata has undergone manual curation and is entirely searchable in our WebApp. Secondly, Accessibility is guaranteed as the metadata is recoverable and made open access, promoting a culture of transparency and shared knowledge. The third point, Interoperability, is upheld by employing knowledge representation and standardized vocabularies that are consistent with international data management practices rather than merely adhering to the FAIR principles. Lastly, the Reusability of our data is ensured as the metadata, which has been manually curated in our work, comprises relevant attributes that conform to the standards accepted by the broader community.

## Conclusions

Our work facilitates the reuse of metagenomics data, providing a WebApp with tools to search for information in a user-friendly way. Furthermore, it allows downloading metadata and metagenomes through our Download Tool. In different parts of this manuscript, we pointed out potential ways scientists interested in animal-associated metagenomes could reuse the data present in our database in new studies. It is relevant to indicate that, in most cases, further, targeted metadata would be necessary for such studies. The AnimalAssociatedDB may raise awareness of the importance of rich metadata to the reusability of metagenomic data. Besides being a valuable tool for scientists studying animal microbiomes and conducting meta-analyses, we shed light on the current bias in the data in public repositories toward vertebrates, temperate regions, and animals used in livestock, medical research, and pets. This work demonstrates that more studies on animal microbiomes outside these fields are necessary (e.g., studies involving biodiversity-, conservation-, and biotechnology-oriented surveys). Moreover, our study highlights the need for more research in underexplored geographic regions of the globe (e.g., tropical areas; Fig. 5), which contain most of the animal biodiversity on Earth and hold an untapped potential in their associated microbiomes.

### Availability and requirements

#### Project name

AnimalAssociatedMetagenomeDB.

**Project home page**: https://webapp.ufz.de/aamdb/.

#### Operating system(s)

Platform independent.

#### Programming language

R, Python.

#### Other requirements

Python3.

#### License

GNU GPL v3.

#### Any restrictions to use by non-academics

See License.

### Electronic supplementary material

Below is the link to the electronic supplementary material.

Supplementary Material 1

Supplementary Material 2

Supplementary Material 3

Supplementary Material 4

Supplementary Material 5

Supplementary Material 6

## Data Availability

The dataset used during the current study is available in the SRA [http://www.ncbi.nlm.nih.gov/Traces/sra] and MG-RAST [http://metagenomics.anl.gov/] repositories.
